# Dual Role for Pld1 in Klebsiella pneumoniae Virulence: Transcriptomics and Proteomics Provide Insights into Direct and Indirect Effects

**DOI:** 10.1021/acs.jproteome.4c01146

**Published:** 2025-05-21

**Authors:** Mayara de Mattos Lacerda de Carvalho, Talyta Soares do Nascimento, Gustavo Miranda Rocha, Livia Carvalho Barbosa, Paulo Mascarello Bisch, Cedric Delporte, Pierre van Antwerpen, Jean-Marie Ruysschaert, Paulo Ricardo Batista, Leticia Miranda Santos Lery

**Affiliations:** † Laboratório de Microbiologia Celular, 196605 Instituto Oswaldo Cruz, Fundação Oswaldo Cruz, Rio de Janeiro 21040-900, Brazil; ‡ Unidade de Pesquisa Urogenital, Centro Biomédico, Departamento de Anatomia, 28130 Universidade do Estado do Rio de Janeiro, Rio de Janeiro 0551-030, Brazil; § Programa de Computação Científica, Fundação Oswaldo Cruz, Rio de Janeiro 21040-900, Brazil; ∥ Laboratório de Física-Biológica, Instituto de Biofísica Carlos Chagas Filho, 28125 Universidade Federal do Rio de Janeiro, Rio de Janeiro 21941-902, Brazil; ⊥ RD3-Pharmacognosy, Bioanalysis and Drug Discovery and Analytical Platform, Faculty of Pharmacy, Université Libre de Bruxelles, 1050 Brussels, Belgium; # Structure and Function of Biological Membranes Laboratory, 26659 Universite′ Libre de Bruxelles, 1050 Brussels, Belgium

**Keywords:** phospholipase D, capsule, fimbriae, bacterial pathogenesis

## Abstract

Klebsiella pneumoniae is an opportunistic pathogen frequently found in healthcare settings, exhibiting resistance to carbapenems and third-generation cephalosporins. Hypervirulent community-acquired strains are also emerging. According to the World Health Organization (WHO), it is the top priority for developing new treatment strategies. A putative phospholipase D (PLD1) was linked to K. pneumoniae virulence, as a mutant strain is avirulent in a mouse model. However, the PLD1 function remains unclear. In the current study, no interaction between PLD1 and lipids was detected in a fat-blot. Lipidomic profile was not altered between strains or infected cells. To shed light on the role of PLD1, we compared the gene expression profile of a wild-type x *pld1* mutant and found 330 modulated genes. Noteworthy, capsular polysaccharide genes were increased in the wild-type, while the mutant expressed higher levels of fimbriae, conjugation systems, and stress-protection proteins. Electron microscopy confirmed a loose capsule in the mutant, which also showed an enhanced adhesion to epithelial cells. A pulldown experiment using PLD1 as bait identified 48 macrophage proteins as putative ligands, including ribosomal, RNA-related, small GTPases, and cytoskeleton-related proteins. It suggests that PLD1 may modulate host cell complexes, favoring the infection. These findings provide novel clues about PLD1′s role in virulence, guiding further investigations.

## Graphical abstract

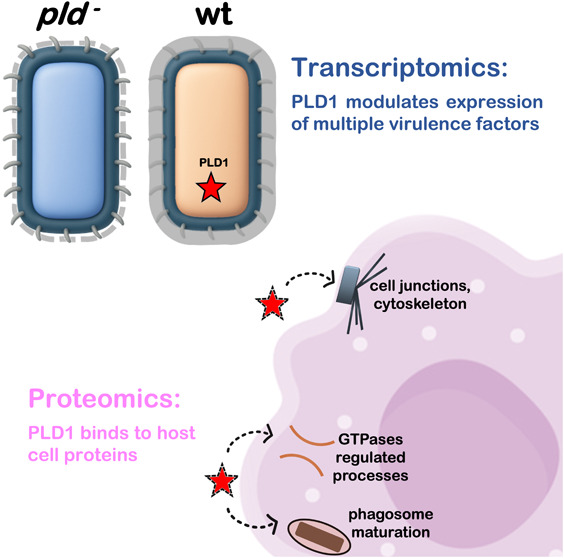

## Introduction

Klebsiella pneumoniae is a ubiquitous enterobacterium causing opportunistic infections in both human and nonhuman animals.^1^ It is well-known in nosocomial environments, carrying genes that confer resistance to multiple antimicrobials, including carbapenems and third-generation cephalosporins—the frontline drugs for treating multidrug-resistant bacteria.^2–4^ However, community-acquired infections also have gained prominence due to hypervirulent phenotypes.^5,6^ The World Health Organization has included this species at the top of the priority list for developing new therapeutical approaches.^7^ Research on bacterial virulence mechanisms and their regulation may contribute to developing alternative disease control methods and holds significance for global public health.

Bacterial phospholipases are enzymes that hydrolyze phospholipids, key components of cell membranes and signaling molecules. Therefore, phospholipases may perform critical roles in membrane remodeling, release of bioactive lipids, and be involved in bacterial virulence and pathogenesis.^8,9^ Phospholipases (PLs) may be classified from A to D, according to the position of hydrolysis of the catalyzed reaction. Phospholipases D (PLD) usually hydrolyze the bond between the phosphate group and the headgroup, forming phosphatidic acid (PA) and a free headgroup. A catalytic domain with a conserved HxKxxxxD motif is required for such enzymatic activity, as it interacts with the substrate, facilitating the cleavage of the phosphodiester bond.^10^

This reaction plays a critical role in various physiological processes, including the structural integrity and fluidity of the bacterial membrane, the production of secondary messengers (such as PA) that regulate stress responses, and biofilm formation. Secreted phospholipases may also be involved in the degradation of extracellular components, disruption of host cell membranes, or interfering with organelles trafficking and signaling cascades, thus promoting infection and weakening immune responses.^9^ Due to its involvement in bacterial pathogenesis, PLD represents a potential target for novel antimicrobial therapies. Inhibition of PLD activity may reduce bacterial virulence and biofilm formation, rendering bacteria more susceptible to immune clearance and antimicrobial treatment.

It has been shown that K. pneumoniae HS11286 translocates Tle1^KP^, an active membrane-targeting phospholipase effector, through a type VI secretion system (T6SS).^11^ Tle1^KP^ is implicated in intraspecies and interspecies competition. Moreover, it was described that the K. pneumoniae Kp52.145 strain encodes putative phospholipase D (PLD1) within a T6SS genomic locus. A transposon mutant interrupting the *pld1* gene is avirulent in a mouse pneumonia model.^12^

In the current study, to verify if PLD1 interacts with lipids, we performed a fat-blot. No interaction between PLD1 and the 15 lipid molecules tested was detected. In addition, a similar lipidomic profile was observed in wild-type x *pld1* mutant bacteria, as well as macrophages infected with them. To shed light on the role of PLD1, we performed a multiomic approach. We compared the gene expression profile of wild-type x *pld1* mutant bacteria by RNA-Seq. The results indicate that a mutation in the *pld1* gene modulates the expression of 330 genes in the phospholipid domain of Kp52.145. Capsular polysaccharide genes were upregulated in the wild-type strain. Accordingly, we detected a loose capsule in the *pld1* mutant strain using electron microscopy. Furthermore, in response to the mutation in the *pld1* gene, K. pneumoniae increases the expression of genes related to fimbria, conjugation systems, and proteins involved in cellular stress protection. Moreover, we found that the disruption of the *pld1* gene enhances bacterial adhesion in the Caco-2 intestinal epithelial cells.

To identify PLD1 targets in eukaryotic cells, we performed a pulldown experiment using beads coated with recombinant PLD1, incubated these beads with THP-1 monocyte extracts, and identified PLD1 ligands by mass spectrometry. The PLD1 interactome revealed 48 proteins from a human macrophage extract, including proteins related to the cellular junctions, cytoskeleton, lipids, endocytosis, and phagocytosis processes. In conclusion, this study provides novel clues about the role of PLD1 in the virulence of Kp52.145, paving the way for further investigations.

## Materials and Methods

### Protein Sequence Analysis

The PLD1 protein sequence is under accession code WP_046043546 in the RefSeq database. Conserved domains were identified with Pfam 34.0. Similarity searches were performed using the NCBI Blastp tool.

### Bacterial Culture

The wild-type K. pneumoniae strain Kp52.145 and its *pld1* ^–^ mutant^12^ were obtained from the collection of the Pasteur Institute. Strains were kept in glycerol 10% in liquid nitrogen for long-term storage and on agar plates for up to a month. Isolated colonies were grown in LB Miller broth medium and incubated at 180 rpm at 37 °C for 16 h. Then, they were spiked 1:200 in LB medium or LB supplemented with 85 mM KCl and incubated at 180 rpm, 37 °C. The *pld1* ^–^ mutant is resistant to kanamycin; thus, it was grown in agar or broth medium supplemented with 50 μg/mL kanamycin.

### Lipidomics

Lipids were extracted as previously described.^13^ In summary, cell pellets were resuspended in 1 mL of water, and then deuterated standards of phosphatidylethanolamine (PE), phosphatidylglycerol (PG), phosphatidylcholine (PC), and cardiolipin (CL) or the Splash Lipidomax Standard mixture (Avanti) were added to achieve a final concentration of 0.02 mg/mL. For each milliliter of the suspension, 3.75 mL of a chloroform and methanol solution (1:2) was added, followed by the addition of 1.25 mL of chloroform and then 1.25 mL of water, with vigorous shaking after each addition. The suspension was centrifuged at 16,000*g* for 20 min at 4 °C, and the lower phase was transferred to another tube and then dried with nitrogen gas. The dried lipids were resuspended in 200 μL of a chloroform/isopropyl alcohol mixture (1:4). Ten microliters of each sample was injected in a rapid resolution liquid chromatography (LC) system (1200 series from Agilent Technologies) fitted with a Zorbax XDB Eclipse Plus column (C18, 4.6 mm × 50 mm, 1.8 μm particle size). The run was 30 min long with the following characteristics: flow rate, 0.3 mL/min; column temperature, 40 °C. Mobile phase A was 0.1% formic acid (positive MS analysis) or 5 mM ammonium acetate, pH 5 (negative MS analysis), and mobile phase B was an isopropanol gradient, which started with 90% solvent A, directly increased to 20% in 10 min, stayed at 20% solvent A for 15 min, and was reequilibrated to starting conditions in 5 min. A 6520 series electrospray ion source (ESI)-quadrupole time-of-flight (QTOF) high-resolution mass spectrometer (Agilent Technologies) was used. For the MS/MS analyses, auto-MS/MS mode was used, and the parameters were as follows: positive or negative mode; high-resolution acquisition mode (4 GHz); gas temperature, 330 °C; drying gas, 7 L/min; nebulizer pressure, 50 psi g; capillary voltage, −4500 V; fragmentor, 210 V; fixed collision energy, 25 eV; MS scan range and rate, 100–1700 *m*/*z* at four spectra per second; MS/MS scan range and rate, 50–1700 *m*/*z* at three spectra per second; auto-MS/MS, three maximum precursors; precursor absolute threshold, 200 counts; active exclusion on two repeats and released after 0.5 min. Data was acquired by Mass Hunter Acquisition for TOF and QTOF version B.04 SP3 (Agilent Technologies). For quantification, single MS analyses were performed with the following parameters: positive or negative mode; extended dynamic range mode (2 GHz); gas temperature, 330 °C; drying gas, 7 L/min; nebulizer pressure, 50 psi g; capillary voltage, 4500 V; fragmentor, 210 V; MS scan range and rate, 100–1700 *m*/*z* at two spectra per second. Data was acquired by Mass Hunter Acquisition for TOF and QTOF version B.04 SP3. The program used for spectrum analysis and database searching was an Agilent Mass Hunter (Agilent Technologies). The database used for the general search was obtained from http://www.lipidmaps.org/, and lipids containing ornithine (OLs) were identified from an in-house database created for this purpose.

### Fat-Blot Assay

A recombinant PLD1 protein was obtained from GenScript, at 0.6 mg/mL in PBS pH 7.4 with 10% de glycerol and 0.5 M de l-arginine, and the purity was estimated at >90%. Membrane lipid strip 2pK P-6002 from Echelon is a hydrophobic membrane containing 100 pmol of triglycerides, phosphatidylinositol, phosphatidylinositol (4)-phosphate, phosphatidylinositol (4,5)-biphosphate, phosphatidylinositol (3,4,5)-triphosphate, phosphatidylserine, phosphatidylethanolamine, phosphatidic acid, diacylglycerol, cholesterol, phosphatidylcholine, sphingomyelin, phosphatidylglycerol, 3-*O*-sulfogalactosylceramide, and cardiolipin. As controls, 1 μL of secondary antibody (Pierce Goat antirabbit IgG HRP conjugated no. 31460) and 4 μL of PLD1 protein (0.6 mg/mL) were spotted directly into the membrane.

The membrane was incubated in PBS at pH 7.2 + 0.1% Tween20 + 0.3% BSA at 4 °C overnight. Then, it was incubated with a 2 μg/uL PLD1 solution in PBS pH 7.2 + 0.1% Tween20 + 0.3% BSA, for 1 h at room temperature. The membrane was washed 3× for 10 min each in PBS pH 7.2 + 0.1% Tween20. Primary antibody anti-PLD1 (Fastbio) diluted 1:200 in PBS at pH 7.2 + 0.1% Tween20 was added for 1 h at room temperature with gentle agitation. The membrane was washed 3× for 10 min each in PBS pH 7.2 + 0.1% Tween20. Secondary antibody antirabbit IgG conjugated to HRP (Pierce Goat antirabbit IgG HRP conjugated n° 31460) in PBS pH 7.2 + 0.1% Tween20 was added for 1 h at room temperature. The membrane was washed 3× for 10 min each in PBS pH 7.2 + 0.1% Tween20 and sensitized with an Amersham ECL Western Blotting Detection Reagent (GE Healthcare).

### RNA Extraction and RNaseq

Overnight cultures of K. pneumoniae wild-type and *pld1* mutant strains were replicated into fresh LB broth containing KCl 85 mM for 4 h at 37 °C and 180 rpm. Three independent growths were performed (biological triplicates). Four milliliters of each bacterial culture was collected by centrifugation for 10 min at 10,000 rpm, and bacterial RNA stabilized in 1 mL RNA later (Thermo Fischer). RNA extraction was performed with an RNeasy Mini Kit (Qiagen). Samples were treated with Turbo DNase (2 U/μL) according to the manufacturer’s instructions (Life Technologies) to eliminate residual DNA, followed by cleaning up with RNeasy Mini Kit (Qiagen). Samples were quantified in nanodrops, and their integrity was evaluated by agarose gel electrophoresis. The rRNA depletion was performed with the Ribo-Zero rRNA Removal Kit (Illumina) and library construction was performed with the TruSeq Stranded mRNA Sample Preparation Kit (Illumina) according to the manufacturer’s recommendations. The final libraries obtained were assessed for quality using a Bioanalyzer (Agilent) and quantified using a Qubit instrument (Thermo Fisher). Sequencing was performed on an Illumina HiSeq Rapid SBS Kit v2 (200 Cycle) and HiSeq Rapid PE Flow Cell v2 at the Plataforma de Sequenciamento de cidos Nucleicos de Nova Geração–RPT01J at the Fundação Oswaldo Cruz (Rio de Janeiro, Brazil).

Raw sequencing read files (bcl files) were converted to fastq files with the bcl2fastq software version 2.17 (Illumina). Technical and low-quality sequences were removed using Trimmomatic (v 2.2.0). Read quality was assessed using FastQC (Babraham Bioinformatics), and filtered reads were mapped with Salmon to the chromosome of K. pneumoniae Kp52.145 (GenBank Accession Nos. FO834906.1, FO834904.1, and FO834905.1) as reference. DESeq2 was used to compare global gene expression between conditions. Genes that showed a differential expression of 2-fold with *p* < 0.05 between strains were considered significantly regulated. Genes were functionally annotated in the Kegg database.

### Bacterial Adhesion and Internalization Assay in Caco-2 Cells

Caco-2, a cell line derived from a patient with colorectal adenocarcinoma, was routinely cultured in Dulbecco’s Modified Eagle Medium (DMEM) (Gibco) supplemented with 10% FBS (fetal bovine serum) (Cripion) in 5% CO_2_ at 37 °C.

A total of 1 × 10^4^ Caco-2 cells were plated in 96-well plates (Kasvi) in High Glucose DMEM + 10% FBS. The plates were incubated at 37 °C and 5% CO_2_ for 16 h for cell adhesion. Bacteria were grown in 5 mL of LB medium supplemented with antibiotics when necessary and harvested in the exponential phase. A cell suspension was prepared in High Glucose DMEM without FBS, and the multiplicity of infection (MOI) used was 50:1. Plates were centrifuged to synchronize infection at 200*g* for 5 min. For the adhesion assay, plates were incubated at 37 °C and 5% CO_2_ for 1 h. Then, the cells were washed three times with PBS to remove nonadherent bacteria. Next, the cells were lysed with 5% saponin, and the lysate containing the bacteria was serial-diluted and plated into LB-agar to count bacteria associated with the cells. For the internalization assay, after 1 h of infection, cells were incubated with High Glucose DMEM containing gentamicin (100 μg/mL) to kill extracellular bacteria at 37 °C and 5% CO_2_ for 1.5 h. After the incubation period, the cells were washed 3 times with PBS and were lysed with 5% saponin to count bacteria inside the cells. All assays were performed in triplicate and repeated 3 times independently. The adhesion was expressed as the total amount of bacteria associated with cells subtracted of the number of bacteria internalized in cells (Adhered bacteria = Associated bacteria – Internalized bacteria).

### Capsule Structure Analysis

Bacterial strains Kp52.145, *wca* ^–^, and *pld* ^–^ were grown to mid-log phase in LB medium, centrifuged at 5000 rpm for 8 min at room temperature, and washed 3 times in PBS. Ten microliters of each 15×-concentrated bacterial suspension was pipetted onto copper grids coated with a thin carbon and Formvar film. The remaining liquid was absorbed from the grid using filter paper. Ten microliters of phosphotungstic acid 2% was added for sample contrastation. The grids were dried and analyzed using a Philips Morgagni D268 electron microscope at 80 kV at the Cellular Ultrastructure Laboratory—IBCCF/UFRJ.

### Recombinant PLD1 Protein

*pld1* gene sequence was optimized, synthesized, and cloned into pET30a­(+) with His-tag for protein expression in Escherichia coli BL21­(DE) Star. These steps were performed by Genescript. The PLD1 recombinant protein sequence is

MHHHHHHMTQEDIITPIATVDTRQCMITSPWFVQNTEYSPMPATYKPLVNGEEAFAAVYHAIMNAQKTVDIICWGFQPSMYFIRDGQSLCIGELLCKIAETKKVQVRILGWEMPCNAAGVGGEANLPGKGVIRYKDRKGQSTTDERYAYDRQWFRQYSLSGEWSDHQLKKGQAGIAEIAAAPIAQRQEKLSSLSPLFVGRGFNFLERAEIAYRAANMALDPDISPDTMLTLAGTVTHHQKTVLVDYELPESAVGFVMGHNMLDEYWDTDKHSALFRPGNNMDPRLGANGKLPRQDISSRVTGPILEHLHHNFAMAWEKETGQDLLTIRDSVSIAKKLKLRALHGTPVMAQLLRTQAQAGKHDIETLYLQAVNNATQFIYIENQYFRWPPLAELINQVAERQSKVGRELHLFVVTNVTDEGIGAGTVNTQRMLEVLGRANIIPEVTKLRKIGQLSNATFGGSVGYIDPGDINKRNREMSEKIADFKKKADEIQSSEILPEERPGLKVHICSLVAPDSPPEEWVPVYIHSKLMIVNDVFTTHGSANINTRSMRVDSEMNIAHEWSSVTRDLRRRLWNMHTNGRGGQDDPAKAFEEWGYILKENKDLQGTKKNKPVASLIKFFYNKSTLSDLD

### Protein Extraction, Pulldown, and Ligand Identification

Monocytes THP-1 (ATCC cat. no. TIB-202, RRID:CVCL_0006) were routinely grown in RPMI-Roswell Park Memorial Institute Medium 1640 (LGC Biotech) supplemented with 10% SFB (Cripion) in 5% CO_2_ at 37 °C. Then, 10^7^ monocytes were washed in PBS and lysed in RIPA buffer containing protease inhibitors (cOmplete Mini, Roche). Three independent cultures of THP-1 monocytes were used for protein extraction.

Protein concentration was measured by Nanodrop (Thermo Fisher), and 10 μg of each protein extract was subjected to a 12.5% polyacrylamide gel electrophoresis to separate the proteins according to their molecular weight and to check sample integrity. Proteins were stained with a Coomassie Blue R-250.

Latex beads of 3 μm (Sigma) were used as a support for PLD1 protein binding. 1.5 × 10^6^ beads were incubated with 120 μg of the PLD1 protein in 50 mM MES buffer pH 6.1. Samples were kept at 4 °C overnight at room temperature for protein adsorption. Then, samples were centrifuged for 15 min at 5000*g* at 4 °C, and the supernatant was discarded. The pellet containing coated beads was resuspended in 200 mg/mL glycine solution in 50 mM MES pH 6.1 to fulfill putative empty binding sites on the bead′s surface. Beads were then collected by centrifugation for 15 min at 5000*g* at room temperature. As a control, “empty beads” were prepared, which underwent the full procedure described above, but the PLD1 protein solution was replaced by a 200 mg/mL glycine solution in 50 mM MES buffer pH 6.1. Beads were incubated with 150 μg of the THP-1 protein extracts (triplicates), in 50 mM MES buffer pH 6.1, for 3 h, at room temperature, with orbital shaking. The samples were centrifuged for 15 min at 5000*g* at room temperature. The beads were further washed twice with PBS to remove unbound proteins and were collected by centrifugation for 15 min at 5000*g* at room temperature. The samples were immediately processed as described below.

A bottom-up proteomics approach was performed to identify the PLD1 ligands. Pellets containing the beads and bound proteins were resuspended in 50 μL of 50 mM NH_4_HCO_3_ and heated for 10 min at 80 °C to promote protein denaturation. Then, proteins were treated with 100 mM DTT (di-thio-threitol) for 30 min at 60 °C to reduce the disulfide bonds and then with 300 mM IAA (iodoacetamide) for 30 min in the dark, at room temperature for cysteines alkylation. Proteins were digested with Trypsin Gold MS-grade (Promega) 0.2 mg/mL at 37 °C overnight; 5% formic acid was added to interrupt the reaction. The samples were centrifuged for 15 min at 5000*g* at room temperature, and the supernatant containing soluble peptides was collected and dried on a Speed Vac System (THERMO SAVANT’s ISS110) for 50 min. The peptides were resuspended in 20 μL of 0.1% TFA (trifluoroacetic acid) and desalted on C18 ziptips (Millipore) according to the manufacturer’s protocol. The peptides were speed-vac dried for 20 min and resuspended in 20 μL of 3% ACN (acetonitrile) and 0.1% TFA.

The peptides were analyzed on LC-MS/MS to assign unique peptide sequences and perform protein identification at the Mass Spectrometry Platform (RPT02A) at the Oswaldo Cruz Institute/Fiocruz. Peptides were desalted in a precolumn Acclaim PepMap 100 C18 (2 cm length, 75 μm internal diameter, 100 Å pore size, and 3 μm particle size) and then separated throughout a 5–50% acetonitrile gradient on a C18 column (Reprosil-Pur packed into a capillary column of 15 cm length, 75 μm internal diameter, 120 Å pore size, 1,9 μm particle size). The Q-Exactive HF-X (Thermo) mass spectrometry apparatus was used for data acquisition. All data were processed using the Xcalibur (version 2.5, Thermo) where the mass/charge values of each precursor (MS) and fragment (MS/MS) were determined from the mass spectra from these chromatograms.

Protein identification and quantification were performed with Pattern Lab V (version 5.00.113). The human proteome at Uniprot (UP000005640), its reverse sequences, and 123 sequences of possible contaminants were used as a database. The decoy (reverse) sequences were used to calculate the FDR (false discovery rate), and the threshold was set to 1%. The identification (peptide spectrum matching, PSM) was carried out considering 3 biological replicates, with 2 technical replicates. The parameters used were High–High, and it was assumed that all cysteines underwent carbamidomethylation, methionines may have undergone oxidation, and tyrosines, serines, and threonines may have been phosphorylated. Trypsin was assigned. The identified proteins were exported into a PLP (Pattern Lab Project) Technical Replicate file, considering only proteins with at least one unique peptide. The presence of proteins in at least 2 biological replicates of the same group were considered, listing proteins identified only in one group (exclusive proteins) and those identified in both groups. The probability mode (0.05) and stringent mode were used. Contaminants and nonspecific hits according to CRAPOME 2.0 database (accessed on October 16, 2023) were excluded. The protein lists were downloaded, and a search was performed in the databases using the ID MAPPING tool from Uniprot, considering the human proteome. Then, gene ontology was used to relate the identified proteins, primarily based on their biological processes, molecular functions, and cellular compartments. String and Cytoscape were used to identify protein–protein interactions networks.

## Results and Discussion

### Computational Analysis

PLD1 (WP_046043546.1) is encoded in locus BN49_RS18775 of the K. pneumoniae Kp52.145 genome. It is a 623 amino acids long protein. Cls and PLDc_SF domains were detected in an analysis in the NCBI Conserved Domain Database, which revealed that PLD1 belongs to the superfamily Cls (COG1502: Phosphatidylserine/phosphatidylglycerophosphate/cardiolipin synthase; Figure 1A) with an *e*-value of 9.33^*e*–23^. Proteins of this superfamily are usually involved in phospholipid biosynthesis. In addition, the catalytic domain of phospholipase D superfamily proteins (PLDc_SF, cl15239) was detected between positions 349 and 552 with an *e*-value of 8.82^*e*–26^ and partially detected between positions 40 and 107 with an *e*-value of 8.78^*e*–11^. This domain architecture (Cls and PLDc_SF domain-containing proteins) is currently found in at least 1383 proteins, according to the NCBI Sparcle Protein Families Models.

**Figure 1:**
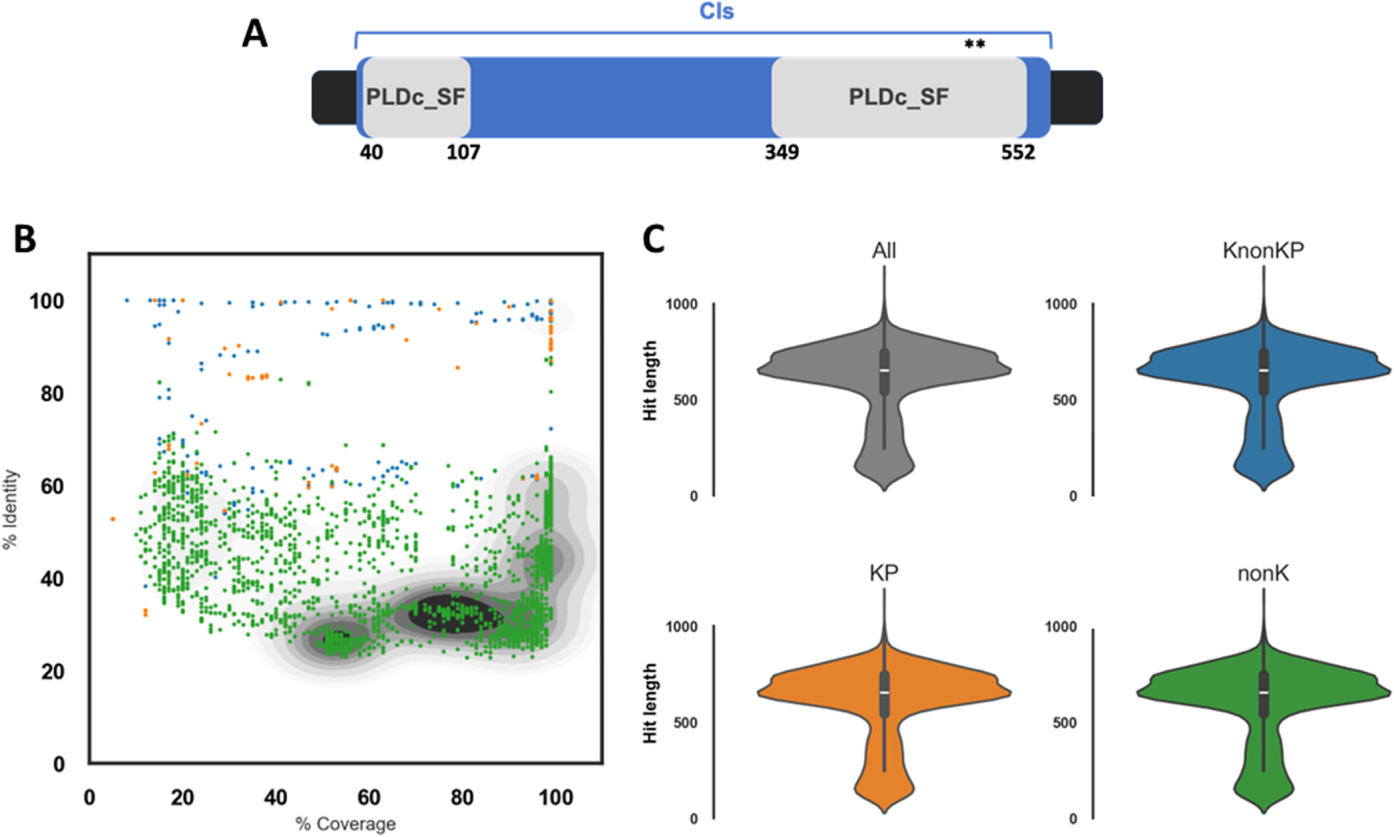
PLD1 domains structure and diversity. (A) Schematic representation of the PLD1 sequence (black rectangle), displaying the region characteristic of the Cls superfamily (blue rectangle: COG1502: Phosphatidylserine/phosphatidylglycerophosphate/cardiolipin synthase) and domains of phospholipase D superfamily proteins (gray rectangles: PLDc_SF, cl15239). (B) Density map: each dot represents a protein hit in the database, according to % identity and % coverage. Orange dots indicate hits from K. pneumoniae strains, blue dots are from *Klebsiella* species other than K. pneumoniae, and green dots are hits from genera other than *Klebsiella*. (C) Violin plots show the distribution of sequences similar to PLD1 according to the hit length. Wider sections of the violin plot indicate a larger number of observations for a given value.

It is worth noting that in another K. pneumoniae strain, the HS11286, Tle1 protein was described as a T6SS lipase effector.^11^ PLD1 and Tle1 are putative lipases. Tle1 has been shown to be an effector of T6SS, while PLD1 is encoded within a T6SS *locus*. Since these are likewise observations, one could wonder if they are related. The analysis we performed showed that Tle1 does not share the same domains nor belongs to the same protein superfamily as PLD1. Tle1 belongs to the α/β hydrolase fold superfamily.

PLD1 is related to K. pneumoniae virulence as a mutant was not able to cause infection in a pneumoniae mouse model, according to Lery et al.^12^ Therefore, it might be involved in Kp52.145 pathogenesis. Considering the high genetic diversity among K. pneumoniae and aiming to understand whether PLD1 would be a very specific effector or could be implicated in the virulence of a larger number of strains, we searched the NCBI database for protein sequences similar to PLD1 (WP_046043546.1). We found 379 hits among K. pneumoniae entries (orange in Figure 1B,C). Among those, 234 hits presented more than 500 amino acids; thus, they look similar to PLD1 in size, as well. Accordingly, most orange dots are on the upper right side of Figure 1B graph, meaning they are highly similar to PLD1 (Figure 1).

Noteworthy, sequences similar to PLD1 in *Klebsiella* species other than K. pneumoniae (KnonKp, blue dots) and also in species outside the *Klebsiella* genus (nonK, green dots) were identified. Accordingly, most blue dots (KnonKp) are in the upper region of Figure 1B graph, while green dots (nonK) are spread around the middle region of the graph. In addition, violin plots (Figure 1C) show the highest density of PLD1 hits over 80% coverage (>500 amino acids), regardless of the species.

Overall, these computational analyses show us that there are several bacteria encoding PLD1 or PLD1-like sequences; therefore, studying the role of this protein may contribute to the understanding of a wide range of strains.

### PLD1 Might Not Be Involved in Lipid Remodeling

As shown above, there are many hits similar to those of PLD1. However, PLD1 is not very similar to well-characterized proteins. Its molecular function is still not known. Considering the sequencing analysis and protein annotation (COG1502: phosphatidylserine/phosphatidylglycerophosphate/cardiolipin synthase), we hypothesized it could be directly involved in phospholipid remodeling. Thus, we performed a fat-blot assay to check whether a recombinant PLD1 protein could bind to 15 different lipids including phosphatidic acid (PA), phosphatidylserine (PS), phosphatidylglycerol (PG), and cardiolipin (CL). We found that PLD1 does not bind to any of them under the conditions tested (Figure 2).

**Figure 2:**
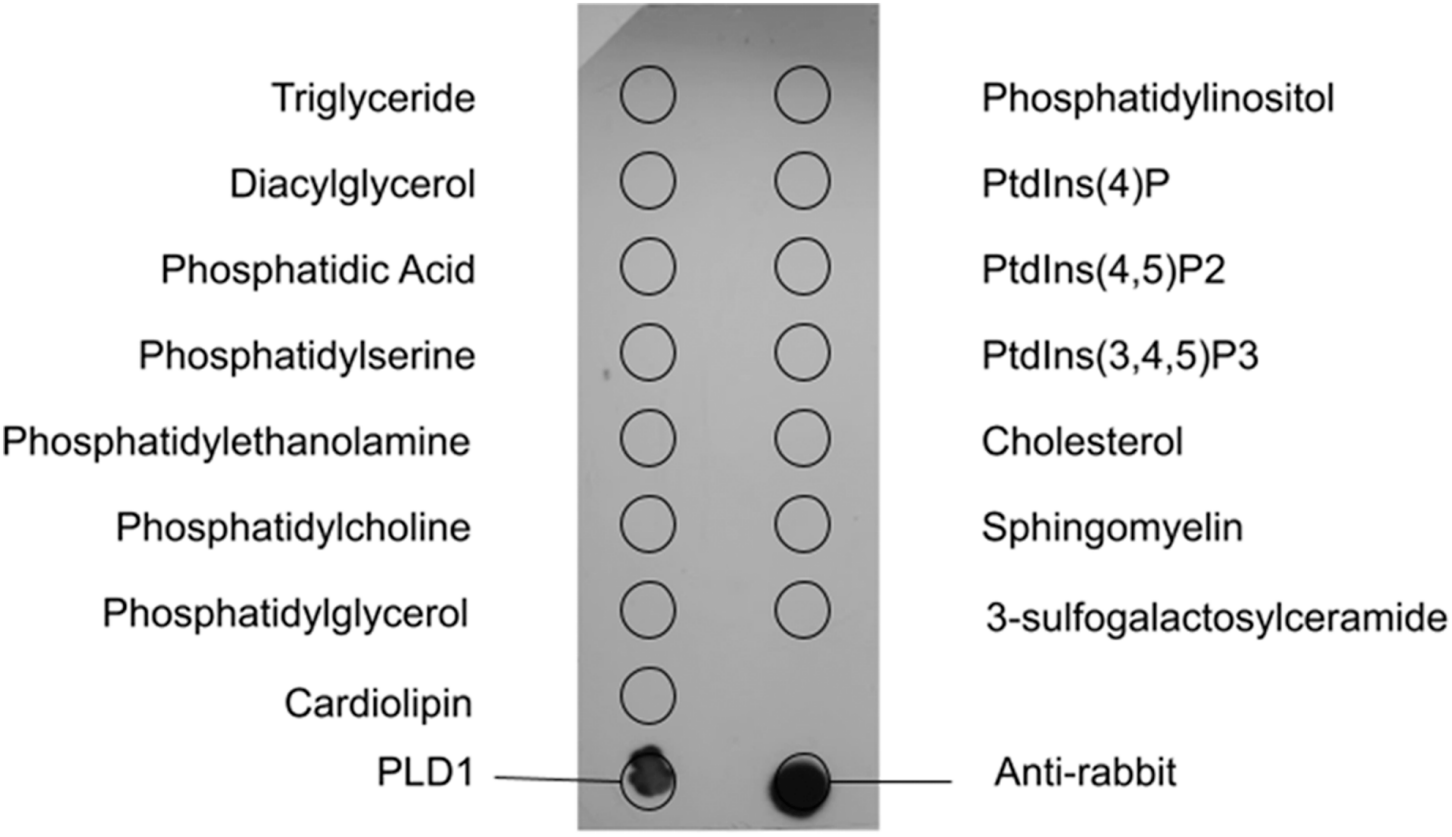
PLD1 does not bind to lipids. Fat-blot assay: the membrane containing immobilized lipids (positions indicated in the image above) was incubated with the PLD1 recombinant protein and further revealed by immunoblotting using the PLD1-specific antibody. As controls, PLD1 and the secondary antibody were spotted in the indicated spaces of the membrane. No interactions were detected between PLD1 and any of the lipids.

This result was unexpected. We hypothesized that PLD1 may not present the expected activity, or the recombinant His-tagged protein is not properly folded and functional under the conditions tested. To further evaluate if it could be an involvement of PLD1 in the modulation of lipid composition, a mass spectrometry-based lipidomic analysis of wild-type versus *pld1* mutant showed that both strains presented similar composition of PE, PG, and CL, as well as acyl chains of similar size and saturation (Figure S1). Despite these unchanged profiles, few molecular species were differentially expressed between strains. There were 2 PE, 3 PG, and 8 CL differentially abundant (Figures S3 and S4). However, as there are no previous descriptions of phospholipase activities acting on the modulation of acyl chains or on such specific molecular species, we did not consider them as directly related to the putative phospholipase activity.

Macrophages are key players of the innate immune system, one of the initial lines of defense that the bacteria may encounter in the host.^14^ Then, we analyzed the lipidomic profile of RAW 264.7 macrophages and compared noninfected cells to cells infected with the wild-type or *pld1* mutant strains for 4 h (Figure S2). We did not find significant differences among lipid classes.

A possible explanation for the lipidomics set of data is that K. pneumoniae may encode redundant enzymes that somehow compensate for the phenotype. It is supported by previously published data^12^ showing changes in the lipidomic profile from the E. coli SD9 expressing PLD1 compared to the parental SD9 strain. SD9 is deficient in phosphatidylserine and cardiolipin, thus presenting a simpler lipidic composition, possibly with less interference by additional factors. Therefore, we do not rule out the possibility that PLD1 may play a role in lipid remodeling; however, under the conditions tested, we did not find evidence for that. Alternatively, we wondered whether PLD1 could indirectly affect bacterial virulence.

### *pld1* Mutant Strain Differentially Expresses Virulence Factors

To have a broad and unbiased view of the effect of the *pld1* mutation on bacterial physiology, we performed RNaseq analysis, comparing wild-type and mutant strains. Overall, reads were mapped to 4741 genes in the K. pneumoniae Kp 52.145 genome, and 330 were modulated, considering differentially expressed genes presenting *p* < 0.05 and fold-change > 2. Of those, 136 genes were highly expressed in the mutant strain, while 194 genes were higher in the wild-type strain (Figure 3A and Supporting Table 1).

**Figure 3:**
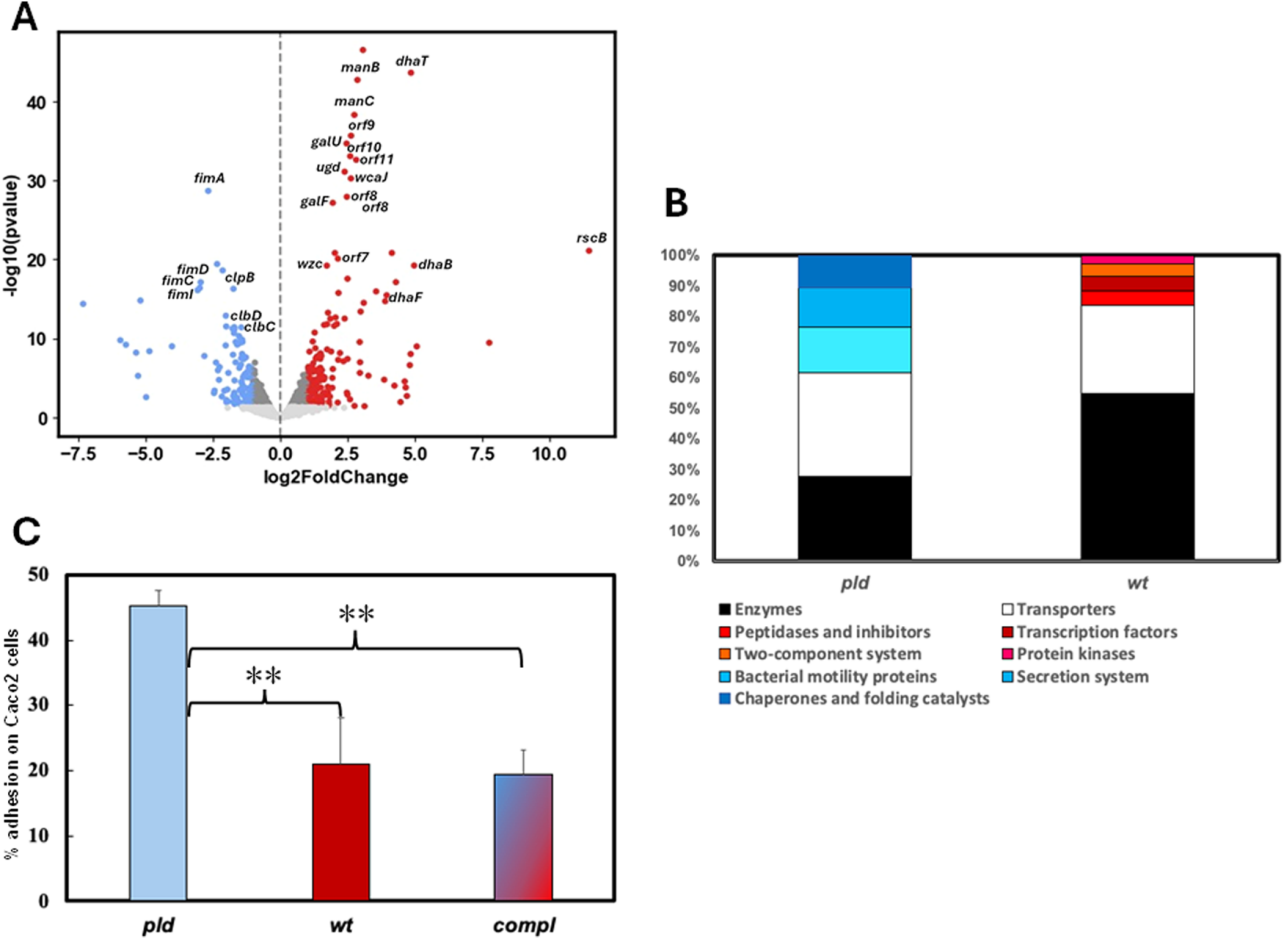
Differential gene expression between K. pneumoniae wild-type and *pld1* ^‑^ and associated phenotype. (A) Volcano plot highlighting differentially expressed genes between K. pneumoniae wild-type and *pld1* ^–^. Genes were considered differentially expressed if *p* < 0.05 and fold-change > 2. Genes with increased expression in the mutant strain are colored in blue, while genes higher in the wild-type strain are shown in red. Genes whose expression was not modulated are plotted in gray. Some genes discussed in the text had their names pointed out in the figure. (B) Differentially expressed genes were categorized using the Kegg Brite hierarchical classification system. The percentage of assigned genes in each strain is shown relative to their associated terms. (C) Bacterial adhesion assay in Caco-2 cells: Infection was performed at a multiplicity of infection (MOI) 50:1, at 37 °C, and 5% CO_2_, for 1 h. Then, nonadherent bacteria were washed and removed. Bacteria adhered to cells were counted by plating serial dilutions into LB agar, after cell lyses with 5% saponin. “compl” refers to the *pld1* mutant strain complemented with a plasmid expressing the *pld1* gene. **p < 0.05.

Differentially expressed genes were classified using the Kegg–Brito system (Figure 3B). Most of them were enzymes or transporters. Apart from those, genes highly expressed in the mutant strain were associated with bacterial motility (7), secretion systems (6), or chaperones and folding catalysts (5). Conversely, genes highly expressed in the wild-type strain encoded peptidases and inhibitors (5), transcription factors (5), two-component systems (4) and kinases (3).

Interestingly, it is noteworthy that several genes encoded in K. pneumoniae virulence *loci* were modulated. For instance, the *pld1* mutant expressed higher amounts of type I adhesin genes, colibactin, and aerobactin synthesis genes. The fimbrial genes *fimA*, *fimC*, *fimD*, *fimF*, *fimG*, *fimH*, and *fimI* were upregulated in the mutant strain. Studies have shown that type 1 fimbria is involved in the attachment and biofilm formation *in vitro*.^15,16^ To verify whether gene regulation reflected on phenotypic variation, we performed a bacterial adhesion assay in intestinal epithelial cells from lineage Caco-2. K. pneumoniae is typically an extracellular bacterium that sometimes may adhere and invade epithelial cells to transcellularly translocate and then disseminate.^17,18^ For this reason, we chose an epithelial cell line for the adhesion assay. The *pld1* ^*‑*^ mutant strain adhered >3× higher than the wild-type strain (Figure 3C). Increased adhesion may contribute to enhanced colonization or persistence.^19^ On the other hand, Fim proteins are antigenic,^20^ eliciting a strong humoral antibody response. Moreover, immunization with FimG protects against K. pneumoniae infection in a mouse model.^21^ Therefore, increased expression of type I fimbria may also result in an increased immune response and infection resolution.

The mutant strain also presented increased levels of colibactin synthesis-related genes, such as *clbB, clbC, clbD, clbF, clbG, clbH, clbI, and clbJ*. Colibactin is a toxin encoded in some K. pneumoniae isolates, usually hypervirulent strains. It alkylates, induces cross-links and double-strand breaks in DNA, and thus may lead to a G2/M cell cycle arrest and a cytotoxic effect in mammalian cells.^22^ Three (*iucA, iucB*, and *iucC*) out of 4 aerobactin synthesis genes presented increased expression in the *pld1* mutant. Aerobactin is a siderophore that confers competitive advantages to K. pneumoniae under iron-limiting conditions.^23^

Besides those classical virulence-related genes, another set of genes highly expressed by the *pld1* mutant is related to ethanolamine metabolism: *eutA, eutB, eutC, eutD, eutE, eutG, eutH, eutJ, eutK, eutM*, and *eutP*. Phosphatidylethanolamine (PE) is one of the most abundant components of cellular membranes and within the gastrointestinal tract, and may be reused in some metabolic pathways. Phosphodiesterases hydrolyze PE into glycerol and ethanolamine (EA), and ethanolamine utilization proteins (encoded in *eut* genes) convert EA into ammonia and acetaldehyde, further used as nitrogen and carbon sources. The EA metabolism has been identified as a critical driver of K. pneumoniae establishment in the gut.^24^ As previously described, K. pneumoniae may be encountered as a commensal bacterium in the gut, or it may behave as an opportunistic pathogen in other tissues. Considering the overexpression of the *eut locus* in the mutant strain, we infer that this mutant behaves like a commensal, while the expression of a functional PLD1 drives K. pneumoniae to an increased virulence state.

On the other hand, the mutant strain presented lower expression of the genes of the capsule operon (*manB, manC, wza, wzb, wzc, orf2, orf7, orf8, orf9, orf10, orf11, orf12, galU, galF, ugd, wcaJ*), as well as its transcriptional regulators *rmpA, rcsA*, and *rscB*. Capsule is recognized as one of the main virulence factors of K. pneumoniae.^25–28^

The *rmp*A (regulator of the mucoid phenotype A) is an activator of capsule (*cps*) *locus* transcription, increasing the virulence of the strain in a mouse model.^29,30^ At least in a particular strain, RmpA acts in an RcsB-dependent manner and is regulated by the availability of iron.^30^ The *rcsB* gene presented the highest log_2_-fold change (>10). It is a positive regulator of *Klebsiella* K2 capsule production,^31^ contributing to capsule regulation through the modulation of the *rmpA* promoter and through additional mechanisms.^32^ Moreover, RcsB plays a key role in K. pneumoniae biofilm formation^33^ and positively regulates the acid stress response.^34^ RcsA is an auxiliary regulator in the Rcs phosphorelay system, interacting directly with RcsB to promote the transcription of genes for capsule synthesis. Therefore, it is reasonable to hypothesize that increased capsule genes in the wild-type strain are to some extent due to the increased expression of *rcsAB*. Interestingly, it has been previously shown that RcsAB negatively regulates the *fim* gene cluster.^31^ Therefore, *rcsAB* downregulated in the mutant also could explain increased type 1 fimbrial gene expression in the *pld1* strain.

Additionally, the differential expression of several genes encoding membrane or membrane-associated proteins, such as the maltoporin (*lamB2*), maltose/maltodextrin system (*malF, malG, malH, malK, malM, malS*), the porins OmpA, OmpK17 (*ompX*) is noteworthy.

Considering the differential expression of capsule and membrane-associated components, we analyzed bacterial surface structures, especially the polysaccharidic capsule, using electron microscopy (Figure 4). The results show that the wild-type strain presents a dense capsule surrounding the cell (Figure 4A–C). As a control, we analyzed the capsule mutant (*wca*) and clearly no capsule structure is observed (Figure 4D–F). Interestingly, in the *pld1* mutant strain, we observed a capsule-like structure surrounding the cells; however, they are somehow fragmented and loosely associated with the cell surface (Figure 4G–I). Therefore, our data strongly suggest a negative regulation of capsule in the mutant strain and an increase of adhesins. Together, they could partially explain the reduced virulence of the *pld* ^–^ strain.

**Figure 4:**
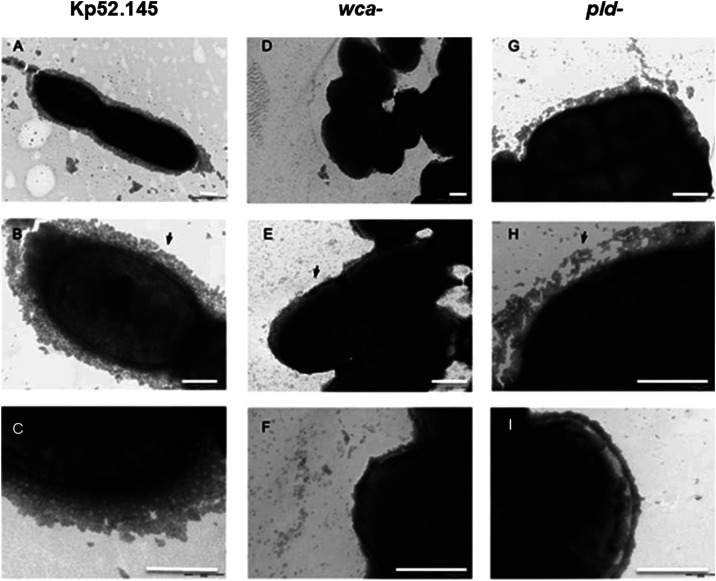
Capsule is differentially organized in the K. pneumoniae *pld1* ^–^ mutant. Electron microscopy of K. pneumoniae Kp 52.145 wild-type (A–C), a capsule mutant (*wca* ^–^, D–F), and the *pld1* ^–^ (G–I) strain. The arrow in panel (B) points to the capsular structure in the wild-type strain. In panel (E) (capsule mutant), no structure surrounding the bacteria was observed. In panel (H), the arrow points to a loose capsular structure in the *pld1* ^*‑*^ mutant strain. The scale bars (white) are 500 nm.

### PLD1 Interacts with Eukaryotic Proteins

Considering that PLD1 might be secreted through T6SS, we speculated whether the PLD1 could act directly on host cells. To gain insights, we incubated protein extracts from THP-1 monocytes with PLD1-covered beads and identified PLD1 interacting partners (Figure 5A). As a control, empty beads were incubated with the same extracts. It should be noted that the approach performed, using cell lysate as a source of ligands, may have a bias toward the identification of proteins that are stable outside of their cellular context. Overall, we identified 112 proteins, 64 proteins both in control and PLD1-covered beads, and 48 exclusively in PLD1 beads (Figure 5B and Supporting Information 2). The mass spectrometry proteomics data have been deposited to the ProteomeXchange Consortium via the PRIDE partner repository with the data set identifier PXD059279.

**Figure 5:**
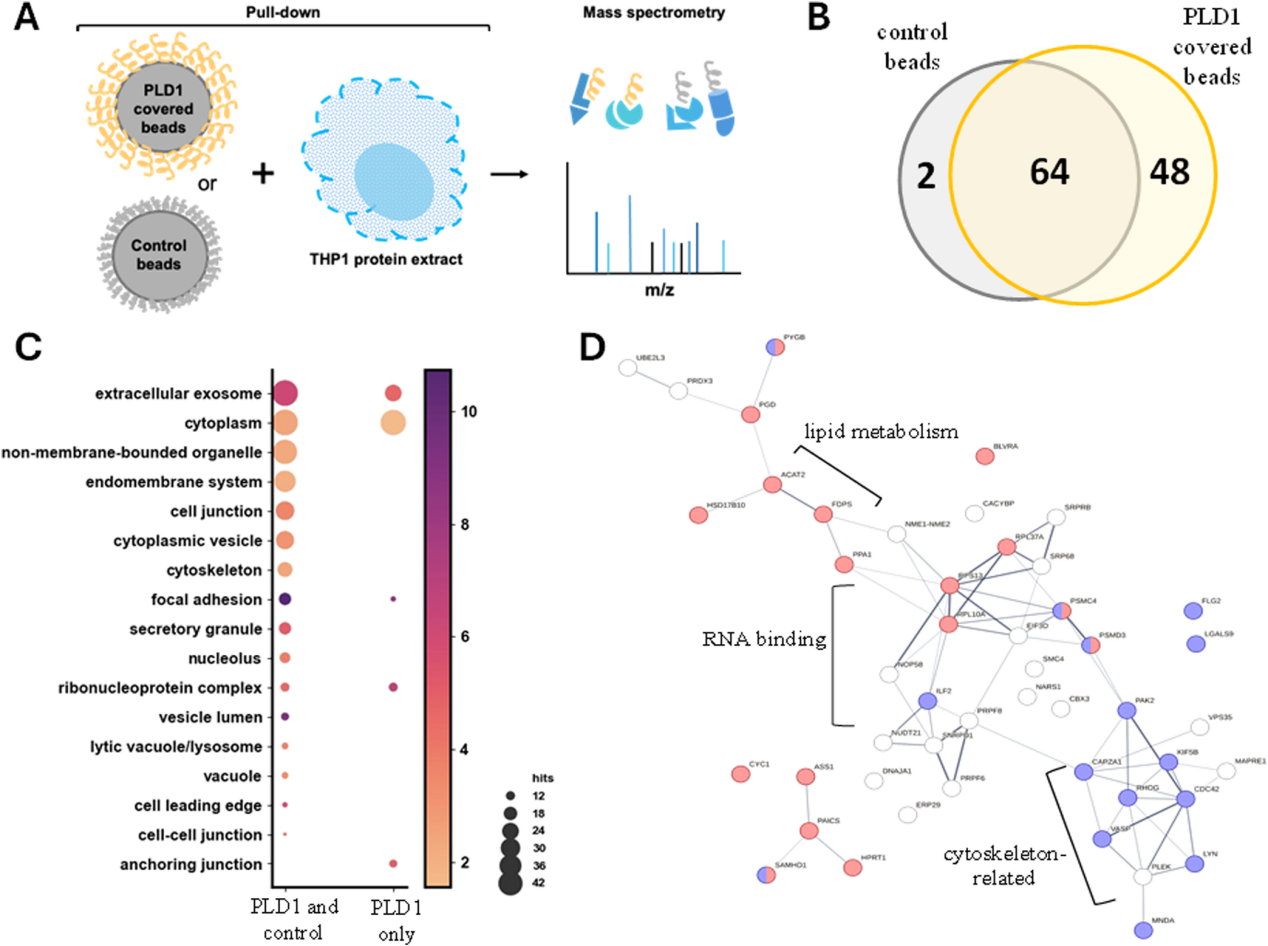
Putative ligands of PLD1 in the host cells. (A) Experimental approach used for ligand identification: beads were covered with PLD1 (yellow) and incubated with an extract of proteins (blue) from the monocytic lineage THP-1. After washing steps, the bound proteins were trypsin-digested and processed for mass spectrometry identification. Empty beads were used as controls (gray). (B) Venn diagram showing that 48 proteins were recovered exclusively from PLD1-covered beads. (C) Gene ontology terms enriched related to proteins identified. The scale bar indicates the enrichment, while the circle sizes are related to the number of hits associated with each term. (D) Protein–protein interactions described in the String database among the 48 proteins exclusively identified from PLD1-covered beads. Proteins annotated as metabolism-related are represented in red, while those associated with the immune system are in blue.

The gene ontology terms enriched in the set of proteins exclusively identified from PLD1-covered beads revealed that most hits are cytoplasmic or related to extracellular exosomes. Moreover, PLD1 may interact with components of anchoring junctions, focal adhesions, and the ribonucleoprotein complex (Figure 5C). Interestingly, disrupting cellular junctions is an important strategy for many pathogens, including Pseudomonas aeruginosa, Helicobacter pylori, and enteropathogenic E. coli. By targeting cell–cell interaction complexes, pathogens take advantage to transmigrate across epithelial barriers.^35^

Among the PLD1 putative ligands related to focal adhesions, we detected: MAPRE1 (microtubule-associated protein RP/EB family member 1), VASP (vasodilator-stimulated phosphoprotein: actin-associated protein involved in cytoskeleton remodeling and cell polarity), CDC42 (cell division control protein 42 homologue: a plasma membrane-associated small GTPase), and RHOG (rho-related GTP-binding protein required for the formation of membrane ruffles during macropinocytosis). Small GTPases are proteins that bind guanosine triphosphate (GTP, active state) and may hydrolyze GTP to guanosine diphosphate (GDP), switching to an inactive state. The GTP or GDP-bound states are involved in the regulation of cytoskeleton remodeling and vesicular trafficking, among others.^36^ Those processes are related for instance to bacterial invasion, phagosome maturation, and bacterial killing or survival.^37–39^

Moreover, additional cytoskeleton-associated proteins were found, such as CAPZA1 (F-actin-capping protein subunit α-1), PAK2 (serine/threonine protein kinase that plays a role in a variety of different signaling pathways including cytoskeleton regulation), and KIF5B (kinesin-1 heavy chain: microtubule-dependent motor), and PLEK (Pleckstrin). This set of proteins is highlighted in Figure 4D.

According to the Reactome pathways database analysis using the String interface, we found that 17 (out of the 48 putative PLD1 ligands) are involved in cell metabolism (Figure 5D, red nodes) and 15 were related to the immune system (blue nodes). Altogether, these data suggest that PLD1 may interact with host cell proteins and modulate immunometabolism in response to an infection. Thus, it partially explains why the *pld*1 mutant is avirulent in a mouse model.

## Conclusions, Limitations, and Perspectives

K. pneumoniae is a prominent pathogen of significant concern mainly due to its association with antimicrobial-resistant infections, including carbapenems and other last-resort antibiotics.^7^ Resistance limits treatment options and increases costs, treatment time, morbidity, and mortality. Augmenting this threat, the emergence of hypervirulent strains raises additional distress. Hypervirulent strains encode supplementary virulence factors that enable them to cause infections, even in healthy individuals. The recent detection of strains simultaneously hypervirulent and multiresistant underscores the need for detailed knowledge of bacterium–host molecular interaction mechanisms.^40–42^ K. pneumoniae virulence factors are usually involved in increased fitness, bacterial survival abilities, and countermeasures to host defenses.^43^

Since 2014 PLD1 is implicated in bacterial pathogenesis, as a mutant strain was avirulent in a mouse pneumonia model.^12^ At that time, it was hypothesized that PLD1 could be involved in lipid metabolism as the heterologous expression of *pld1* in E. coli SD9 modulated the content of PG and CL. SD9 is a strain lacking phosphatidylserine and cardiolipin, resulting in a less complex lipid composition compared to both its parent strain and Kp 52.145. Despite those findings, our group did not detect significant alterations in the lipid composition of K. pneumoniae wild-type versus *pld1* mutant, nor in cells infected with those strains (Figures S1–S4). Moreover, in this work, we have shown that PLD1 does not bind to the lipids tested on a fat-blot assay (Figure 2). We do not rule out the possibility that PLD1 might be involved in lipid metabolism in K. pneumoniae; however, under the conditions tested so far, we have not yet found direct evidence for that.

In contrast, we found that *pld1* mutation impacted the expression of the capsule, a well-known virulence factor of this species,^43^ along with fimbria (Figure 3). We confirmed that gene expression alterations are indeed reflected in phenotypic variations, analyzing the capsule structure by electron microscopy and evaluating bacterial adhesion to eukaryotic cells. Additional genes encoding virulence factors were modulated, such as aerobactin and colibactin. Aerobactin is a siderophore associated with hypervirulent strains.^23^ Colibactin induces interstrand DNA cross-linking in host cells, and thus it is genotoxic.^22^ Those factors are associated with increased fit and virulence.^28,40^ This set of data indicates that, to some extent, the *pld1* avirulence in the mouse model might be a result of indirect effects modulating the expression of other virulence factors.

In contrast, we have found that the recombinant PLD1 can bind to host proteins (Figure 4). The pulldown experiment performed allowed us to recover proteins directly bound to PLD1, but probably also proteins that form complexes to those bound to PLD1. Analyzing the set of 48 putative ligands of PLD1, we found that there are several ribosomal and RNA-related proteins, as well as small GTPases and cytoskeleton-related proteins. A previous study using polarized intestinal epithelial cells has depicted that actin and microtubule cytoskeleton, as well as GTPases were required for K. pneumoniae translocation.^17^ Therefore, we hypothesize that PLD1 may have an additional role in bacterial pathogenesis by modulating host cell complexes, favoring the infection.

Overall, this study has presented new insights into the role of PLD1. We propose that PLD1 may have a dual role in bacterial virulence: (1) modulating the expression of other bacterial virulence factors; (2) modulating host processes. Regardless of those major advances, it is important that further studies are performed in the future, improving the understanding of PLD1’s role in colonization and different types of infection.

## Supplementary Material





## Data Availability

The mass spectrometry proteomics data have been deposited to the ProteomeXchange Consortium via the PRIDE partner repository with the data set identifier PXD059279.

## Notes

Bacterial lipidomic profile (Figure S1); infected cells lipidomic profile (Figure S2); differentially expressed bacterial PE and PG (Figure S3); and differentially expressed bacterial CL (Figure S4) (PDF)
Data from transcriptomic analysis (Table S1) (XLSX)
