# Supplementary material for: Dual Role for Pld1 in Klebsiella pneumoniae Virulence: Transcriptomics and Proteomics Provide Insights into Direct and Indirect Effects
Source: J Proteome Res. 2025 May 21;24(6):2874–84. doi: 10.1021/acs.jproteome.4c01146 (PMC12150317; doi:10.1021/acs.jproteome.4c01146)
Supplement: Supplementary file 1 [file pr4c01146_si_002.pdf]

A dual role for Pld1 in Klebsiella pneumoniae virulence: transcriptomics and proteomics provide insights into direct and indirect effects

Mayara de Mattos Lacerda de Carvalho[1], Talyta Soares do Nascimento[1], Gustavo Miranda Rocha[2], Livia Carvalho Barbosa[4], Paulo Mascarello Bisch[4], Cedric Delporte[5], Pierre van Antwerpen[5], Jean-Marie Ruysschaert[6], Paulo Ricardo Batista[3], Leticia Miranda Santos Lery[1*]

1- Laboratório de Microbiologia Celular, Instituto Oswaldo Cruz, Fundação Oswaldo Cruz, Rio de Janeiro, Brazil. 21040-900.
2- Unidade de Pesquisa Urogenital, Centro Biomédico, Departamento de Anatomia, Universidade do Estado do Rio de Janeiro, Rio de Janeiro, Brazil. 20551-030.
3- Programa de Computação Científica, Fundação Oswaldo Cruz, Rio de Janeiro, Brazil. 21040-900.
4- Laboratório de Física-Biológica, Instituto de Biofísica Carlos Chagas Filho, Universidade Federal do Rio de Janeiro, Rio de Janeiro, Brazil. 21941-902.
5- RD3-Pharmacognosy, Bioanalysis and Drug Discovery and Analytical Platform, Faculty of Pharmacy, Université Libre de Bruxelles, 1050, Brussels, Belgium
6- Structure and Function of Biological Membranes Laboratory, Universite´ Libre de Bruxelles, 1050 Brussels, Belgium

* Corresponding author: leticia.lery@ioc.fiocruz.br

Keywords: phospholipase D, capsule, fimbriae, bacterial pathogenesis

SUPPORTING INFORMATION:
The following supporting information is available free of charge at ACS website http://pubs.acs.org

- Figure S1: bacterial lipidomic profile
- Figure S2: infected cells lipidomic profile
- Figure S3: differentially expressed bacterial PE and PG
- Figure S4: differentially expressed bacterial CL
- Table S1: data from transcriptomic analysis

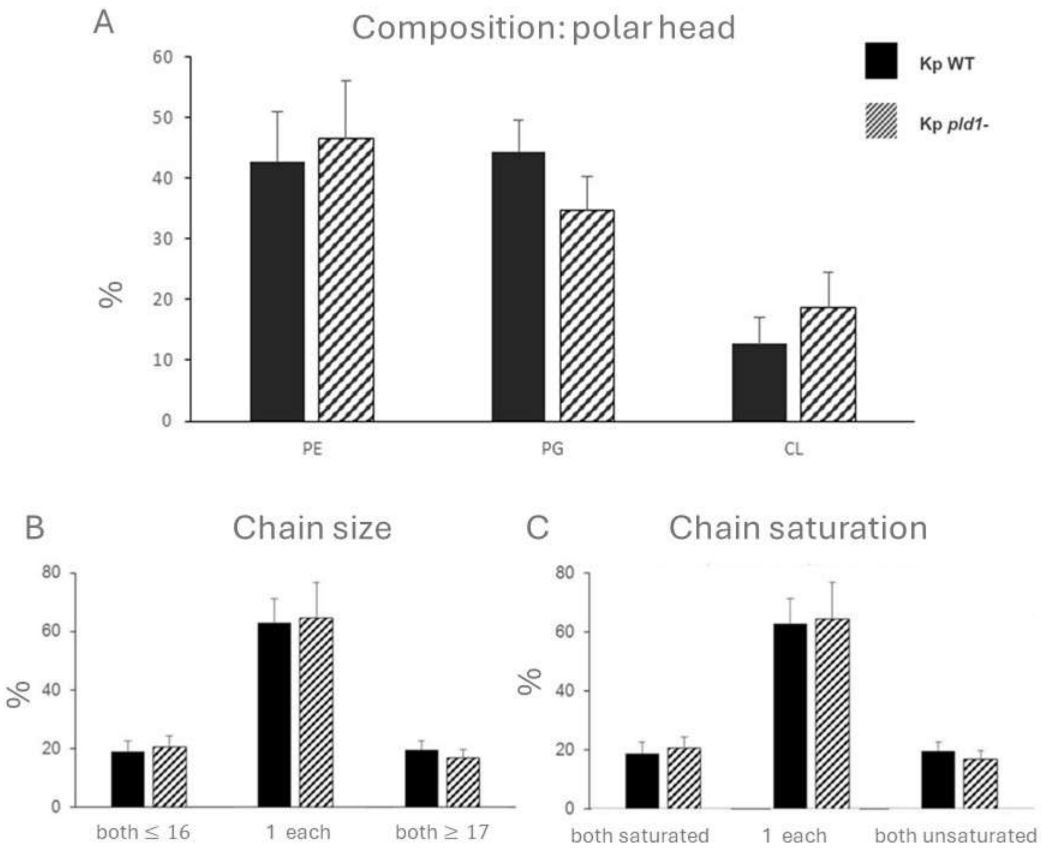

Figure S1: Lipidomic profile of *K. pneumoniae* wild-type and *pld1*⁻ mutant grown in LB for 16 h. Lipids were extracted using Blight and Dyer method, in quintuplicates, and analyzed by LC-MS (C18 – Q-Tof). Quantification was performed using the area under the curve, compared to a standard curve. No differences were observed between the strains concerning the polar heads (A), chain sizes (B), or chain saturation (C).

PE: phosphatidylethanolamine, PG: phosphatidylglycerol, CL: cardiolipin.

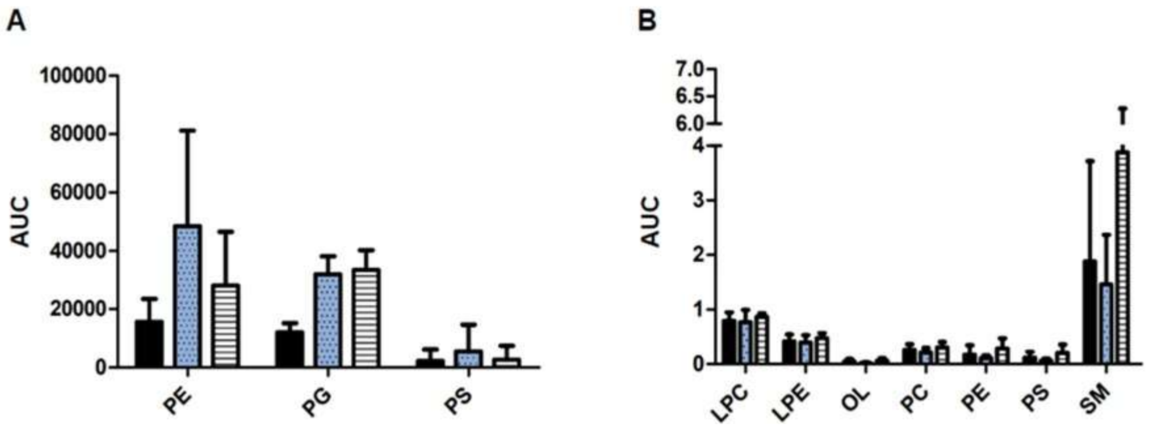

Figure S2: Lipidomic profile of RAW 264.7 macrophages non-infected (black), and infected with *K. pneumoniae* wild-type (blue) or with pld1^{-} mutant (striped) for 4 hours. Lipids were extracted using Blight and Dyer method, in quintuplicates, and analyzed by LC-MS (C18 – Q-Tof). Quantification was performed using the area under the curve, compared to a standard curve. No differences were observed between the samples, nor in negative (A) or positive (B) ion modes.

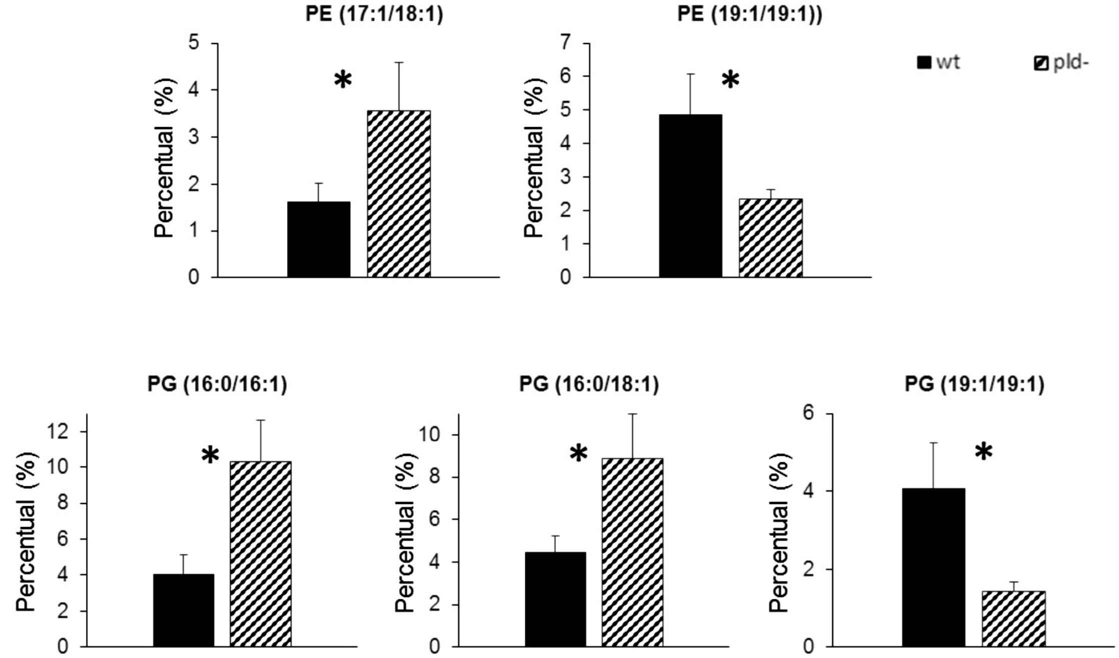

Figure S3: Lipid species differentially abundant between *K. pneumoniae* wild-type and $pld1^{-}$ mutant grown in LB for 16 h. Overall, 26 PE and 19 PG molecular species were identified. From those, 2 PE (17:1/18:1 and 19:1/19:1) and 3 PG (16:0/16:1; 16:0/18:1 and 19:1/19:1) were significant differentially abundant (a * denotes $p<0.05$).

PE: phosphatidylethanolamine, PG: phosphatidylglycerol.

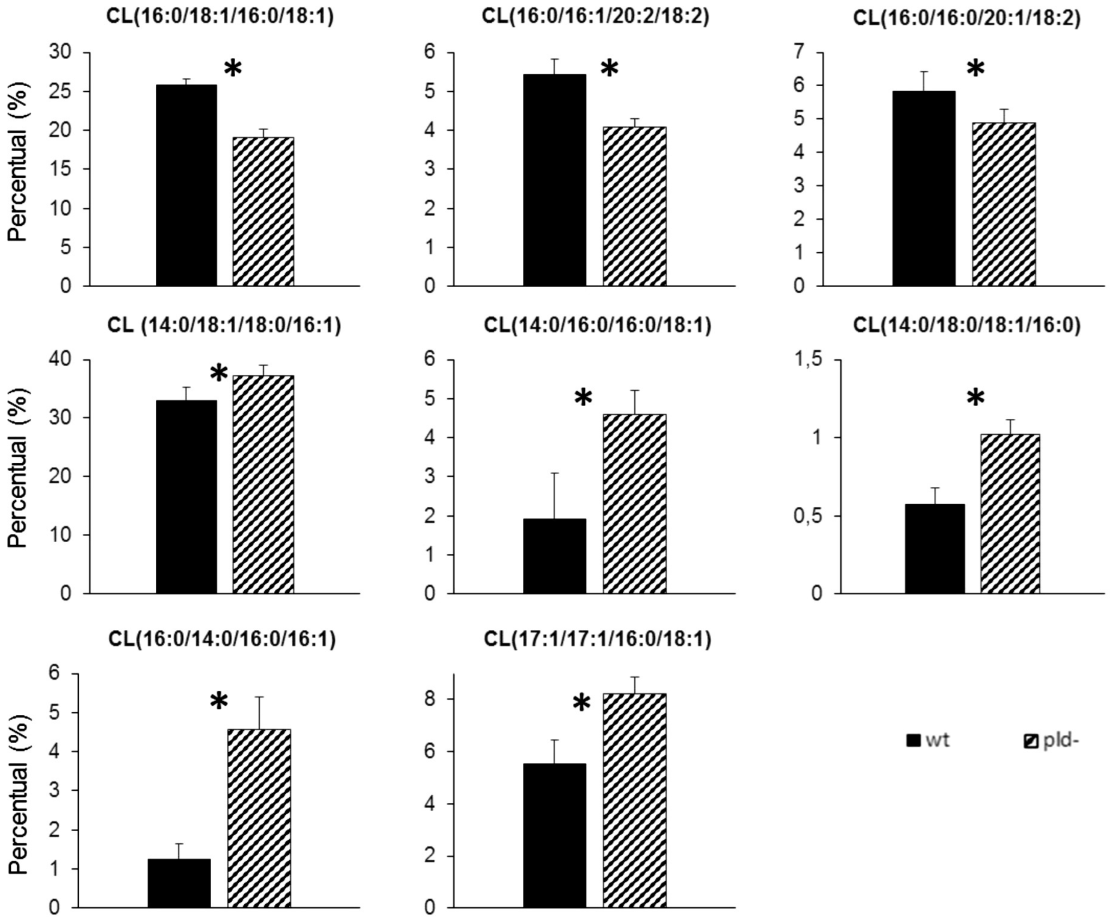

Figure S4: Lipid species differentially abundant between *K. pneumoniae* wild-type and *pld1*$^{-}$ mutant grown in LB for 16 h. Overall, 11 CL molecular species were identified. From those, 8 CL were significant differentially abundant (a * denotes $p<0.05$):

(16:0/18:1/16:0/18:1), (16:0/16:1/20:2/18:2), (16:0/16:0/20:1/18:2), (14:0/18:1/18:0/16:1), (14:0/16:0/16:0/18:1), (14:0/18:0/18:1/16:0), (16:0/14:0/16:0/16:1) and (17:1/17:1/16:0/18:1).

CL: cardiolipin.
